# A patient with severe polytrauma with massive pulmonary contusion and hemorrhage successfully treated with multiple treatment modalities: a case report

**DOI:** 10.1186/s13256-020-02406-9

**Published:** 2020-06-16

**Authors:** Futoshi Nagashima, Satoshi Inoue, Miho Ohta

**Affiliations:** Department of Trauma Surgery and Surgical Critical Care, Saga University, Faculty of Medicine, 5-1-1 Nabeshima, Saga, 849-8501 Japan

**Keywords:** Multiple trauma, Resuscitative endovascular balloon occlusion of the aorta (REBOA), Damage control surgery, Veno-venous extracorporeal membrane oxygenation (VV-ECMO)

## Abstract

**Background:**

The mortality rate is very high for patients with severe multiple trauma with massive pulmonary contusion containing intrapulmonary hemorrhage. Multiple treatment modalities are needed not only for a prevention of cardiac arrest and quick hemostasis against multiple injuries, but also for recovery of oxygenation to save the patient’s life.

**Case presentation:**

A 48-year-old Japanese woman fell down stairs that had a height of approximately 4 m. An X-ray showed pneumothorax, pulmonary contusion in her right lung, and an unstable pelvic fracture. A chest drain was inserted and preperitoneal pelvic packing was performed to control bleeding, performing resuscitative endovascular balloon occlusion of the aorta. A computed tomography scan revealed massive lung contusion in the lower lobe of her right lung, pelvic fractures, and multiple fractures and hematoma in other areas. An emergency thoracotomy was performed, and then we performed wide wedge resection of the injured lung, clamping proximal to suture lines with two Satinsky blood vessel clamps. The vessel clamps were left in the right thoracic cavity. The other hemorrhagic areas were embolized by transcatheter arterial embolization. However, since her respiratory functions deteriorated in the intensive care unit, veno-venous extracorporeal membrane oxygenation was used for lung assist. Planned reoperation under veno-venous extracorporeal membrane oxygenation was performed on day 2. Since her respiratory condition improved gradually, the veno-venous extracorporeal membrane oxygenation circuit was withdrawn on day 7. She was transferred to the psychiatric ward of our hospital on day 75.

**Conclusion:**

Utilizing multiple treatment modalities such as resuscitative endovascular balloon occlusion of the aorta, damage control surgery, transcatheter arterial embolization, and veno-venous extracorporeal membrane oxygenation with appropriate timing saves a patient with severe polytrauma with massive pulmonary contusion including intrapulmonary hemorrhage.

## Background

The mortality of multiple trauma with severe chest trauma of Abbreviated Injury Scale (AIS) > 3 is very high: 15.1% in all ages and 28.4% in those 65 years or older [1]. Quick hemostasis and treatments with appropriate prioritization for injured organs are essential to rescue patients with polytrauma, especially severe truncal trauma with pulmonary contusion with massive hemorrhage. Severe lung contusion can lead to massive hemothorax and severe tracheobronchial bleeding. Emergency surgery is determined based on the chest drainage volume in cases of hemothorax. However, it is relatively difficult to find tracheobronchial bleeding due to positive airway pressure ventilation at the early phase of injury. Massive amounts of tissue factor are released due to the lung contusion, which worsens coagulopathy and results in an increased amount of bleeding. Respiratory dysfunction can also be caused from blood flowing into the normal lung area from the pulmonary contusion area. In such cases, it is very difficult to maintain respiratory function with conventional respiratory management of a respirator only. Therefore, veno-venous extracorporeal membrane oxygenation (VV-ECMO) may be the best option, as well as the last resort, to save those patients’ lives.

Here we report a case of severe polytrauma of a 48-year-old woman with massive pulmonary contusion containing intrapulmonary hemorrhage; we were able to successfully save her life utilizing multiple treatment modalities, including resuscitative endovascular balloon occlusion of the aorta (REBOA), damage control surgery (DCS), transcatheter arterial embolization (TAE), intrabronchial block balloon, and VV-ECMO.

## Case presentation

A 48-year-old Japanese woman fell down stairs that had a height of approximately 4 m. Her family called 119 (a direct-dial emergency number that connects the caller to the fire and emergency medical services) and the fire station simultaneously dispatched a “doctor-helicopter” from our hospital. She had past medical history including cholecystectomy and schizophrenia, and no remarkable family history. Her respiratory rate was 30 breaths/minute and blood oxygen saturation (SpO_2_) was 90% with oxygen at 10 L/minute. Her breath sound in her right chest was diminished. Her pulse rate was 130 beats/minute and her blood pressure was 88/55 mmHg. Her extremities were cold with sweat present, suggesting she was in a shock status. A focused assessment sonography for trauma (FAST) revealed hemoperitoneum in the pelvic space and a hemothorax in the right side of her chest. Her consciousness levels were 12 points (E3, V4, M5) according to the Glasgow Coma Scale at first contact and no coarse paralysis of limbs was observed. She was brought to our hospital by a doctor-helicopter, undergoing initial fluid resuscitation and respiratory assist with a bag valve mask (BVM).

**Fig. 1:**
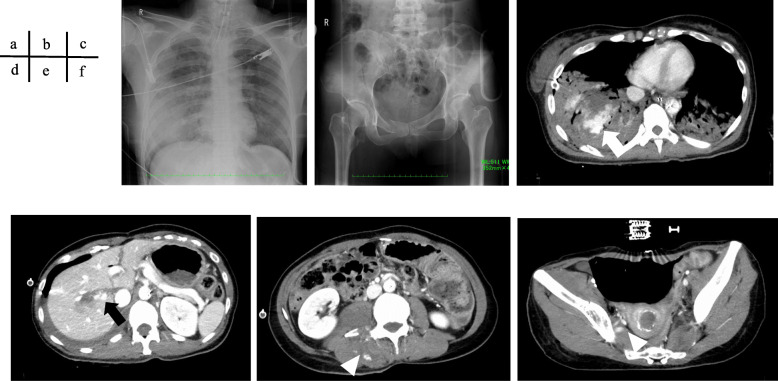
X-ray and computed tomography findings on admission. **a** Chest X-ray showing heavy contusion and hemopneumothorax in the right lung. **b** Pelvis X-ray showed disruption of bilateral sacroiliac articulation and fractures of bilateral pubic bone. **c** A chest computed tomography scan revealed massive lung contusion with major active extravasation (*white arrow*) in the lower lobe of the right lung and moderate lung contusion in the lower lobe of the left lung. **d** Abdominal computed tomography scan revealed liver injuries with extravasation (*black arrow*). **e**, **f** Pelvic computed tomography showed massive hematoma with extravasation (*white arrow head*) in erector spinae muscle and fractures of transverse process of lumbar vertebrae (**e**) and multiple pelvic fractures involving moderate hematoma with extravasation of contrast media in retroperitoneal pelvic space (**f**)

Her hemodynamics deteriorated remarkably with a pulse rate of 120 beats per minute and 50 mmHg systolic blood pressure on arrival. SpO_2_ was below 90% under respiratory assist with BVM. She was given 6 units of type O Rh plus red blood cells (RBC). A 7-French aortic occlusion catheter (Rescue Balloon®, Tokai Medical Products, Aichi, Japan) was inserted from her right femoral artery and was inflated with 20 ml distilled water to maintain her systolic blood pressure above 90 mmHg. A chest X-ray showed pneumothorax and pulmonary contusion in her right lung (Fig. 1a). A pelvis X-ray revealed an unstable fracture (Fig. 1b). The FAST showed a moderate hemothorax in the right side of her chest and a small amount of hemoperitoneum in Morison’s pouch and Douglas pouch. A 28-French chest drain was inserted, and preperitoneal pelvic packing (PPP) was performed to control bleeding from the unstable pelvic fracture, followed by application of a pelvic binder. A whole-body contrast-enhanced computed tomography (CT) scan was performed. The chest CT scan revealed massive lung contusion with major active extravasation of contrast media in the lower lobe of her right lung and moderate lung contusion in the lower lobe of her left lung (Fig. 1c). The abdominal CT revealed liver injuries with extravasation of contrast media, as well as massive hematoma in an erector spinae muscle with extravasation of contrast media and fractures of transverse process of lumbar vertebra (Fig. 1d, e). The pelvic CT confirmed multiple pelvic fractures involving moderate hematoma with extravasation of contrast media in retroperitoneal pelvic space (Fig. 1f).

**Fig. 2:**
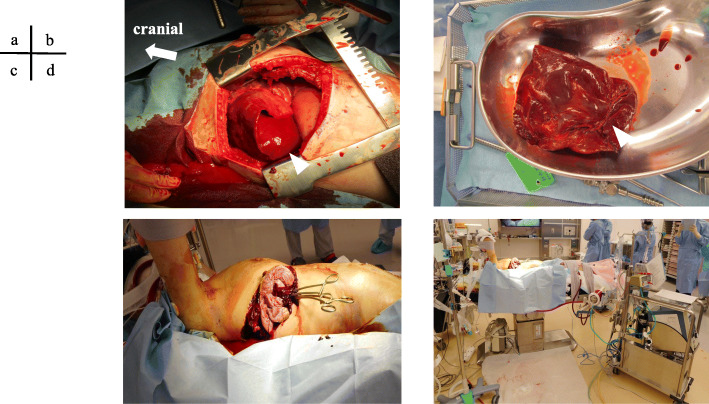
The findings during damage control thoracotomy and views of the surgical site and operation room just before planned reoperation. **a** The lower lobe of right lung is remarkably swollen due to intrapulmonary hemorrhage and hematoma (*white arrow head*). **b** The removed lower lobe of right lung is shown (*white arrow head*). **c** Two vascular clamps were placed at the proximal site of the resected area to avoid unexpected rebleeding. **d** Pulmonary function was well maintained by veno-venous extracorporeal membrane oxygenation during the surgery

The laboratory data on initial arrival are shown in Table 1. The Injury Severity Score (ISS) in this case was 48 and the probability of survival was calculated as 29.1%. We first decided to perform damage control thoracotomy since the right severe pulmonary contusion was thought to be a main bleeding source based on CT. Hemorrhage influx into the lumen of our patient’s trachea from the right pulmonary contusion was observed in a tracheal tube when she returned to the operation room (OR) in our emergency department (ED) from the CT room. A double lumen tracheal tube was replaced with a single lumen tracheal tube to prevent blood influx into healthy lung areas before emergency thoracotomy in a supine position. The amount of bleeding in the right thoracic cavity was approximately 1500 ml. The main sources of bleeding in her chest were the lung contusion area of the lower lobe of her right lung and multiple rib fractures. Intrathoracic packing with surgical gauze was performed as a temporary hemostasis to control bleeding from the sites of fractures of ribs. Since the right lung contusion had extended to near the hilum of lung (Fig. 2a), the hilum of lung was clamped for temporary hemostasis of the lung. At that time, her body temperature was 35.2 °C, base excess and pH of arterial blood gas analysis (BGA) were 10.5 mmol/L and 7.099, respectively, and a persistent oozing of blood from a non-surgical site was recognized. Therefore, we decided to perform wide wedge resection of the lung using a surgical stapling device as a DCS instead of an anatomical lobectomy. We converted the hilum clamp to a limited clamp to the injured lobe with two Satinsky blood vessel clamps. The vessel clamps were left in the right thoracic cavity, clamping proximal to suture lines. Then, therapeutic intrathoracic packing for hemorrhage from multiple rib fractures was performed. Surgical packing gauzes were mainly put in the dorsal and lower side in right thoracic cavity in order to maintain respiratory function of the upper and middle lobes of her right lung, and the vessel clamps were stabilized with additional surgical towels. After the placement of a chest drain, a temporary vacuum packing chest closure was performed and DCS was finished (total surgical time was 55 minutes).

**Table 1:**
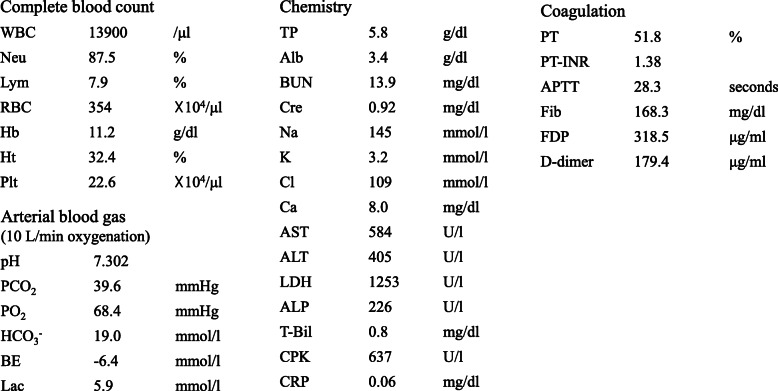
Laboratory data on admission

*Alb* albumin, *ALP* alkaline phosphatase, *ALT* alanine aminotransferase, *APTT* activated partial thromboplastin time, *AST* aspartate aminotransferase, *BE* base excess*, BUN* blood urea nitrogen, *Ca* calcium, *Cl* chloride, *CPK* creatine phosphokinase, *Cre* creatinine, *CRP* C-reactive protein, *FDP* fibrin degradation product, *Fib* fibrinogen, *Hb* hemoglobin*, HCO*_*3*_^*−*^ bicarbonate, *Ht* hematocrit, *K* potassium, *Lac* lactate, *LDH* lactate dehydrogenase, *Lym* lymphocyte, *Na* sodium, *Neu* neutrophil, *PCO*_*2*_ partial pressure of carbon dioxide, *Plt* platelets, *PO*_*2*_ partial pressure of oxygen, *PT* prothrombin time, *PT-INR* prothrombin time-international normalized ratio, *RBC* red blood cells, *T-Bil* total bilirubin, *TP* total protein, *WBC* white blood cells

**Fig. 3:**
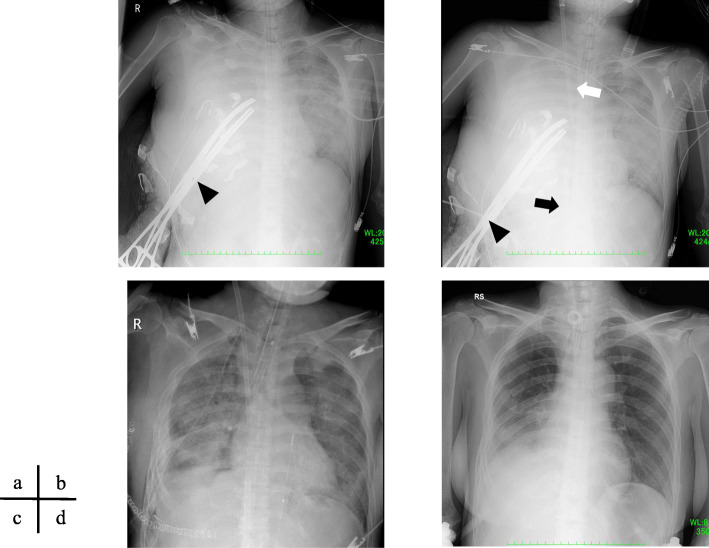
Chest X-ray findings post operation. **a** Chest X-ray findings following damage control surgery (thoracotomy). The bleeding source was clamped (*black arrowhead*) and intrathoracic packing was performed. **b** Chest X-ray findings post establishment of veno-venous extracorporeal membrane oxygenation system. The catheter to establish the veno-venous extracorporeal membrane oxygenation was placed from the right internal jugular vein (feeding catheter, *white arrow*) and right femoral vein (drainage catheter, *black arrow*). **c** Chest X-ray findings after planned reoperation on day 2. **d** Chest X-ray findings on day 15 showed a significant improvement in both lung areas

After the DCS for the chest, TAE was performed for severe liver injuries including medial segment and right lobe with gelatin sponge. Furthermore, her left subcostal artery, left first and fourth lumbar arteries, right first to fourth lumbar arteries, right superior gluteal artery, bilateral iliolumbar arteries, right obturator artery, and left lateral sacral artery were embolized in the same fashion (total procedure time was 118 minutes). Meanwhile, her respiratory status worsened including decreased partial pressure of oxygen in arterial blood (PaO_2_)/fraction of inspired oxygen (FiO_2_) (P/F) ratio and elevation of partial pressure of carbon dioxide (pCO_2_) on arterial blood gas. In the intensive care unit (ICU), her respiratory functions deteriorated with a P/F ratio below 50: pCO_2_ on BGA over 70 mmHg, pH of 7.099, and base deficit of − 12 mmol/L. Since a ventilator was no longer sufficient to maintain her respiratory condition, VV-ECMO was initiated as a lung assist: FiO_2_ 1.0, oxygen flow 2.0 L/minute, and veno-venous (VV) blood flow 4.5〜5.0 L/minute (Fig. 3b). A bronchial block balloon was inserted into her right lower bronchus to reduce pressure to the suture lines of the lung. Blood and clots in the other side of the trachea and bronchus were toileted with a bronchoscope. Her hemodynamics and respiratory function improved gradually with these treatments. A blood transfusion continued to maintain the following: hemoglobin (Hb) > 9.0 g/dl, fibrinogen > 150 mg/dl, and platelet > 10 × 10^4^/μl. The total blood transfusion for 24 hours included 82 units of RBC, 136 units of fresh frozen plasma (FFP), and 70 units of platelet concentrate (PC).

**Fig. 4:**
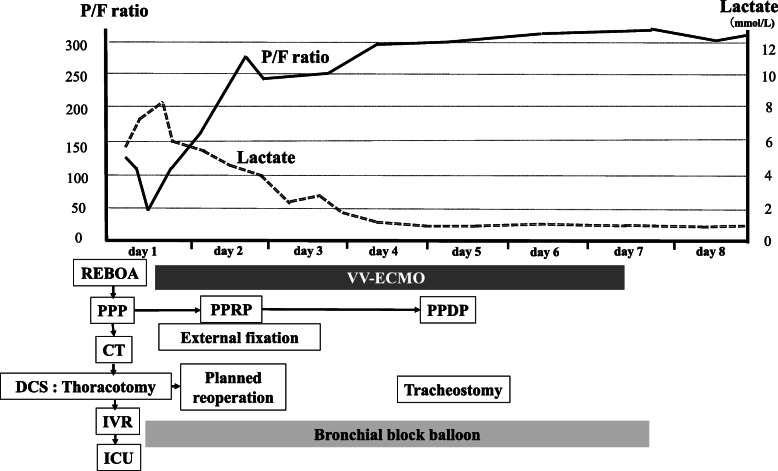
Clinical course and treatment. Elapsed course of intervention and examination were shown with a value of lactate and partial pressure of oxygen in arterial blood/fraction of inspired oxygen ratio. On day 1, preperitoneal pelvic packing was performed introducing resuscitative endovascular balloon occlusion of the aorta as hemostatic treatment strategy. Emergency thoracotomy after computed tomography examination was performed, and transcatheter arterial embolization was sequentially performed to stop the bleeds from multiple injuries. Veno-venous extracorporeal membrane oxygenation was performed because of a deterioration of the patient’s pulmonary function with a partial pressure of oxygen in arterial blood/fraction of inspired oxygen ratio of below 50 just after admission to the intensive care unit. The partial pressure of oxygen in arterial blood/fraction of inspired oxygen ratio was remarkably improved by veno-venous extracorporeal membrane oxygenation, and the patient was successfully weaned off from the veno-venous extracorporeal membrane oxygenation on day 7. There was an obvious inverse correlation between lactate level and partial pressure of oxygen in arterial blood/fraction of inspired oxygen ratio. The lactate value was affected by hypoxia as well as hemorrhagic shock. On day 2, the planned reoperations for the chest and pelvis were performed and the hemorrhages in the thoracic injuries were successfully stopped. Since bleeding from the pelvic fracture could not be fully controlled, preperitoneal pelvic repacking was performed, and packing gauze was removed on day 5. A bronchial block balloon was inserted into the right lower bronchus to protect the stump of lung resection site from collapse due to excess intrabronchial pressure. We performed tracheostomy on day 5 as the patient was required to be on mechanical ventilation for a long period of time. *CT* computed tomography, *DCS* damage control surgery, *ICU* intensive care unit, *IVR* interventional radiology, *P*/*F* partial pressure of oxygen in arterial blood/fraction of inspired oxygen, *PPDP* preperitoneal pelvic depacking, *PPP* preperitoneal pelvic packing, *PPRP* preperitoneal pelvic repacking, *REBOA* resuscitative endovascular balloon occlusion of the aorta, *VV*-*ECMO* veno-venous extracorporeal membrane oxygenation

The planned reoperation for her chest and pelvis under VV-ECMO was performed on day 2 (Fig. 2c, d). When Satinsky blood vessel clamps were cautiously removed, there was a slight oozing of blood from the suture line at the resection site of the lower lobe of her right lung. The vessel forceps were reclamped and the stump was interruptedly sutured with two pairs of Teflon pledgets for hemostasis. We closed her chest with two chest drains placed following additional suture for hemorrhage from the multiple rib fractures area (Fig. 3c). Since slight bleeding continued from the right side of her pelvic retroperitoneal space after removal of PPP gauze, repacking and external fixation for pelvic fracture were also performed. On day 3, RBC and PC were appropriately transfused as our patient’s Hb and platelets were decreased due to VV-ECMO. Her respiratory function was completely dependent on VV-ECMO. Fluid infusion was restricted and a diuretic was administered to make her run on the dry side and her bloody and mucinous phlegm was deliberately removed by a bronchoscope. On day 5, the PPP gauze was removed and the wound was definitively closed. Her respiratory condition improved gradually, and P/F ratio became over 250, and her pCO_2_ level was within the normal limit when FiO_2_ and blood flow of VV-ECMO were decreased. The VV-ECMO circuit was withdrawn and the bronchial block balloon was removed on day 7. Our patient’s clinical course with intervention and examination and the change in lactate levels and P/F ratio until day 8 are shown in Fig. 4. On day 15, her respiratory condition was improved to the desired extent with no need for a ventilator (Fig. 3d). Pneumonia and right intrathoracic infection subsequently occurred and were treated by antibiotics. She needed another 45 days of rehabilitation to be able to walk independently, and was transferred to the psychiatric ward of our hospital on day 75.

## Discussion

The present case was on the verge of cardiac and respiratory arrest due to a serious polytrauma with severe hemorrhagic shock, which required a rapid control of bleeding using a damage control strategy and respiratory management utilizing VV-ECMO.

We determined to control the massive bleeding with REBOA according to FAST and X-ray findings, and control the shock caused by severe hemorrhage. In this case, time from admission to successful temporary aortic occlusion (TAO) with REBOA was only 12 minutes. In the American Association for the Surgery of Trauma (AAST) prospective Aortic Occlusion for Resuscitation in Trauma and Acute Care Surgery (AORTA) registry, TAO was not significantly different in the time between an open aortic occlusion (OAO) and REBOA: median/interquartile range (IQR) of 15.0/70 and 30.5/36 minutes, respectively [2]. Our TAO with REBOA was very short and was performed as quickly as the time it took for OAO. REBOA was placed appropriately and smoothly by experienced trauma surgeons as almost all the things needed for REBOA insertion were well prepared in advance. All required preparations for REBOA, including equipment, staffing, place (OR), and activation of the DCS were ordered from the accident scene by a doctor-helicopter staff member. Utilizing REBOA in combination with PPP is a very powerful strategy to achieve hemostasis for both the artery and vein in patients with severe unstable pelvic fracture, especially when they are performed quickly and timely.

A CT scan revealed the severe pulmonary contusion with massive intrapulmonary hematoma, which probably required surgery, and multiple injuries with extravasations of contrast media on liver, spine, and pelvis, which required TAE. We determined to perform surgery on her chest first because the pulmonary contusion had a great risk of severe intrathoracic and tracheal bleeding, which would lead to severe coagulopathy due to the release of a multitude of tissue factors and subsequent pulmonary failure. We observed a lung laceration reaching to near the pulmonary hilum, as well as other multiple injuries such as the thoracic wall and sites of fractures of ribs that were causing continuous bleeding on the operation. We decided to perform wide wedge resection of the injured lung as a DCS. It is reported that damage control thoracic surgery is suitable for patients with severe chest trauma with physiological derangement [3]. Mortality was increased in an extent-dependent manner for lung resection: pneumonectomy at 62%, lobectomy at 35%, and wedge resection at 22% [4]. Wedge resection can be performed with a simple and quick procedure and is often applied as a DCS. However, wide wedge resection of the injured lung which reaches near hilum of lung, such as in the present case, is a risky procedure. If large blood vessels and bronchus exist in the excision site, a surgical automatic suturing device occasionally cannot seal them completely, which may result in major bleeding and large air leak with traumatic coagulopathy. Furthermore, repair of such bleeding and air leakage from the suture line may be very difficult, because the resection site tends to move into the inner part of the mediastinum. Therefore, we determined to leave the vessel forceps clamped proximal to suture lines in the intrathoracic space until the planned reoperation was performed. The vessel clamps were secured by putting gauze around them so they did not move from their original positions. Packing gauze was also applied to control minor venous bleeding and oozing from multiple sites of fractures of ribs. The chest was temporarily closed by vacuum packing system. Although it has been reported that intrathoracic packing is an effective method of hemostasis for severe chest trauma as a DCS [5, 6], it may also cause respiratory dysfunction and hemodynamic instability. Interestingly, it has been previously demonstrated that the peak airway pressure in vacuum packing closure (VPC) using intrathoracic packing group was lower than that in the definitive thoracic closure group in studies of chest closure of patients who underwent emergent thoracotomy [3, 7, 8]. We found that intrathoracic packing with temporary closure using VPC was very effective in a case of polytrauma which required control of bleeds from multiple sites simultaneously.

In the present case, VV-ECMO was applied for her severe respiratory failure refractory to conventional ventilator support, which was progressed after DCS and TAE. There are some reports regarding application and usage of VV-ECMO in trauma cases [9]. Previous studies demonstrated that survival rate was improved by VV-ECMO when utilized for patients with lung trauma with no signs of improvement of hypoxia and hypercapnia on conventional ventilator [9, 10]. The mean time from injury to initiation of VV-ECMO was 3.2 days (9) and 4.6 days (10) in these reports, whereas time in our case was approximately 8 hours. In these reports, VV-ECMO was performed for trauma-induced acute respiratory distress syndrome (ARDS) that occurred a few days after the injury. It is known that early application of VV-ECMO for patients with severe trauma is highly challenging when bleeding is not fully controlled since an insufficient fluid volume in the patient’s circulation leads to possible VV-ECMO circuit failure, including blood drainage or infusion failure. However, appropriate hemostasis and blood transfusion were performed in our case, and thus VV-ECMO worked perfectly without any specific troubles, and it could also be initiated in the early stage after the injury.

## Conclusion

Utilizing multiple treatment modalities such as REBOA, DCS, TAE, intrabronchial block balloon, and VV-ECMO with appropriate timing saves a patient with severe polytrauma with massive pulmonary contusion including intrapulmonary hemorrhage.

## Data Availability

Not applicable.

## Abbreviations

AAST: American Association for the Surgery of Trauma
REBOA: Resuscitative endovascular balloon occlusion of the aorta
DCS: Damage control surgery
TAE: Transcatheter arterial embolization
VV: Veno-venous
VV-ECMO: Veno-venous extracorporeal membrane oxygenation
ICU: Intensive care unit
IQR: Interquartile range
AIS: Abbreviated Injury Scale
FAST: Focused assessment sonography for trauma
FiO_2_: Fraction of inspired oxygen
BVM: Bag valve mask
pCO_2_: Partial pressure of carbon dioxide
PPP: Preperitoneal pelvic packing
RBC: Red blood cells
SpO_2_: Blood oxygen saturation
CT: Computed tomography
ISS: Injury Severity Score
OR: Operation room
ED: Emergency department
BGA: Blood gas analysis
P/F: Partial pressure of oxygen in arterial blood (PaO_2_)/fraction of inspired oxygen (FiO_2_)
Hb: Hemoglobin
FFP: Fresh frozen plasma
PC: Platelet concentrate
TAO: Temporary aortic occlusion
AORTA: Aortic Occlusion for Resuscitation in Trauma and Acute Care Surgery
OAO: Open aortic occlusion
VPC: Vacuum packing closure
ARDS: Acute respiratory distress syndrome
